# Supplementary material for: Si/SiGe QuBus for single electron information-processing devices with memory and micron-scale connectivity function
Source: Nat Commun. 2024 Mar 14;15:2296. doi: 10.1038/s41467-024-46519-x (PMC10940717; doi:10.1038/s41467-024-46519-x)
Supplement: Supplementary file 1 — Supplementary Information [file 41467_2024_46519_MOESM1_ESM.pdf]

Supplementary Information

Ran Xue,[1] Max Beer,[1] Inga Seidler,[1] Simon Humpohl,[1,2] Jhih-Sian Tu,[3] Stefan Trellenkamp,[3] Tom Struck,[1,2] Hendrik Bluhm,[1,2] and Lars R. Schreiber[1,2]

[1]*JARA-FIT Institute for Quantum Information, Forschungszentrum Jülich GmbH and RWTH Aachen University, Aachen, Germany*

[2]*ARQUE Systems GmbH, 52074 Aachen, Germany*

[3]*Helmholtz Nano Facility (HNF), Forschungszentrum Jülich, Jülich, Germany*

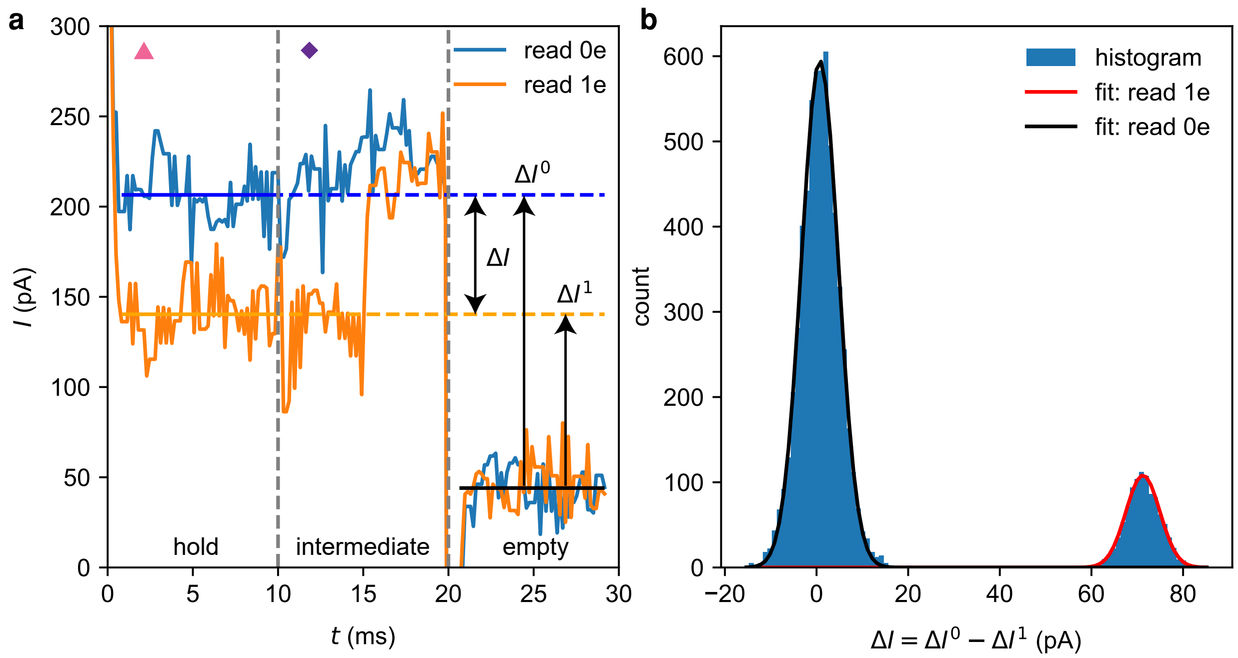

Supplementary Figure 1. **Single-shot charge-readout.** **a**, Two representative SET current traces I recorded during the detection pulse segment D_L. It consists of three stages namely hold, intermediate and empty. Each of them lasts for 10 ms and is labeled by the same symbols in Fig. 1d in the main text. If QD_0 is filled by one and zero electrons, we typically observe the orange and blue traces, respectively. The solid horizontal lines within the hold stage and the empty stage indicate the averaged current levels from which ΔI^0 (arrow) and ΔI^1 (arrow) are calculated. The dashed lines are extended only for guide-to-the-eye. The sensitive operating point of SET is chosen while $V_{S_i}(t = 0)$ are applied to all four clavier gates. After any λ shuttle step, this operation point is recovered despite large electrostatic-cross-coupling of the clavier gates to the SET. **b**, Histogram of current differences ΔI (as defined in panel a). The statistics plotting here is a result of shuttling a single electron for $n\lambda$ where $n = 3$ and $N = 1000$.The x-axis is centered with respect to the averaged current level of detecting an empty QD_0 that is labeled as ΔI^0 in **a**. The two Gaussian fits are assigned to detecting an empty QD_0 (black) and filled QD_0 (red), respectively. The overlap between the two Gaussian fits is relevant to the detection error and is evaluated to 2.29×10^{-20}.

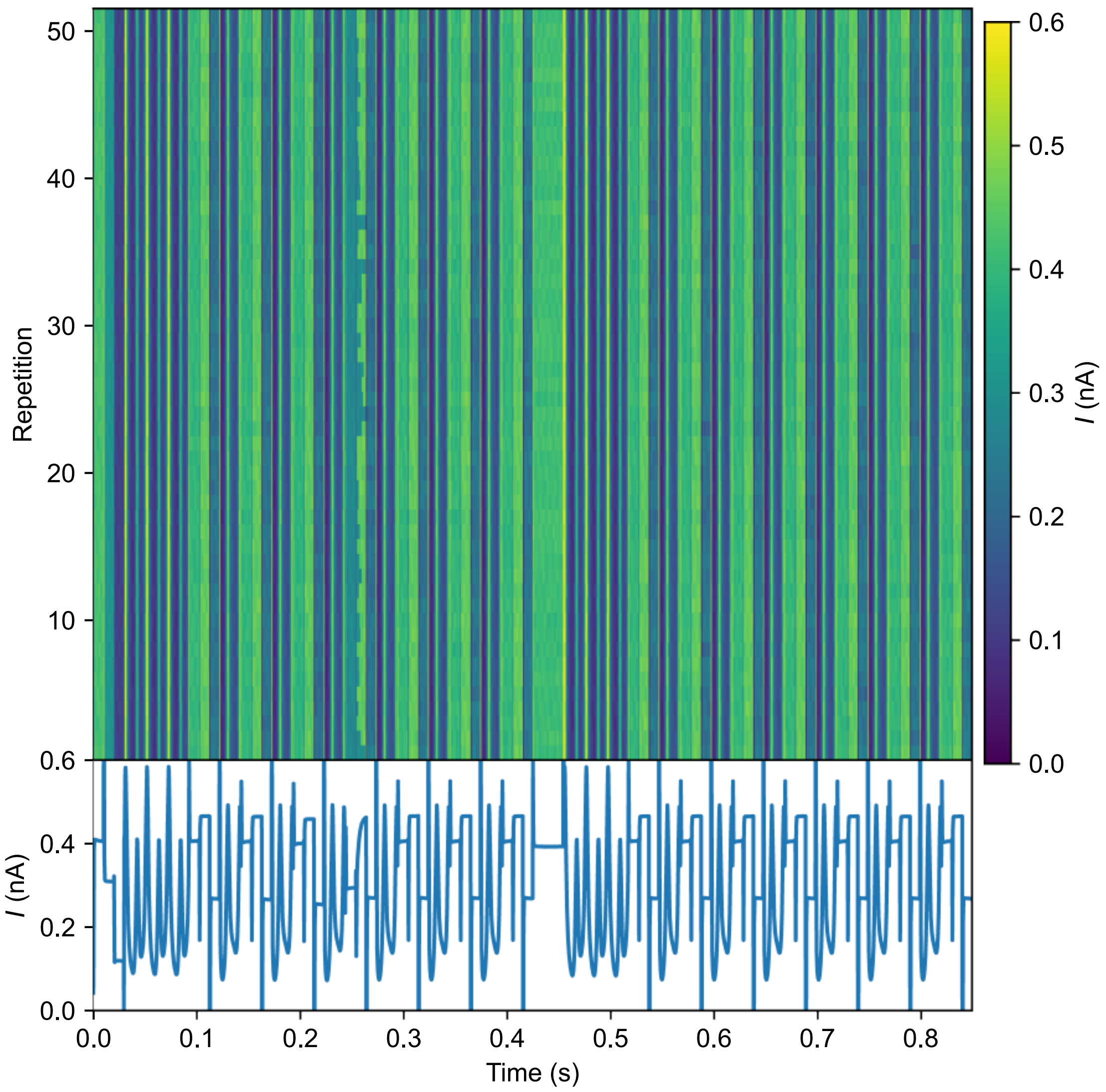

Supplementary Figure 2. **Full SET current traces recorded during the shuttle tomography.** An example of 50 randomly selected repetitions out of 10000 are shown as a false-color plot. The SET current during the single electron shuttle and the reference shuttle with $n = 3$ (Fig. 2a of the main text) are shown. The statistical mean of all 10000 current traces is plotted below the false-color plot.

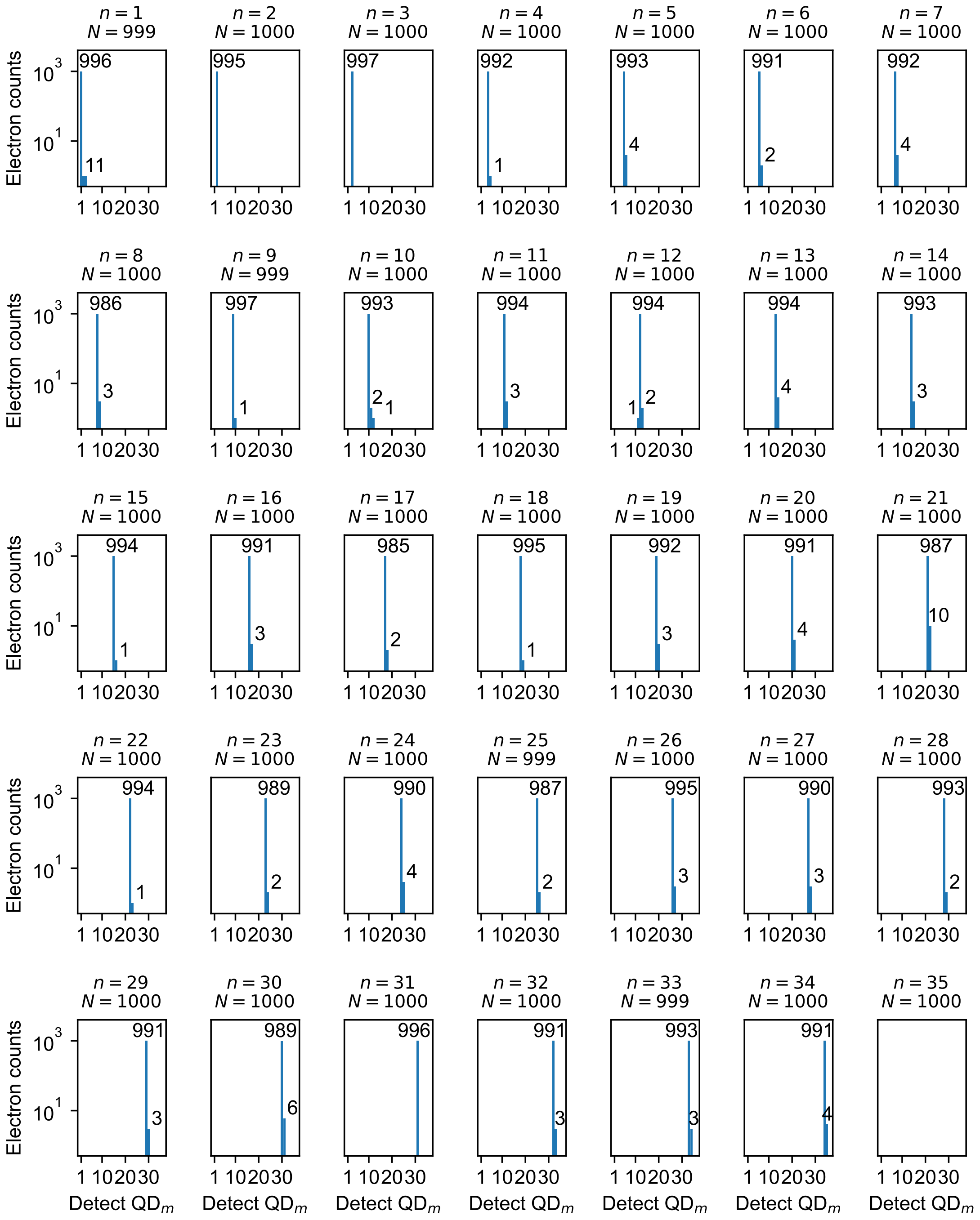

Supplementary Figure 3. **Electron counts for single charge shuttle-tomography.** Each panel is the electron count for N shuttle-pulse repetitions for $A_S = 280\,\text{meV}$ as a function of the QD number m, in which the electrons are detected. For each panel a different n is used in the pulse sequence. Numbers label the height of bars larger than zero. For $n = 35$ the shuttle distance exceeds the physical dimension of QuBus.

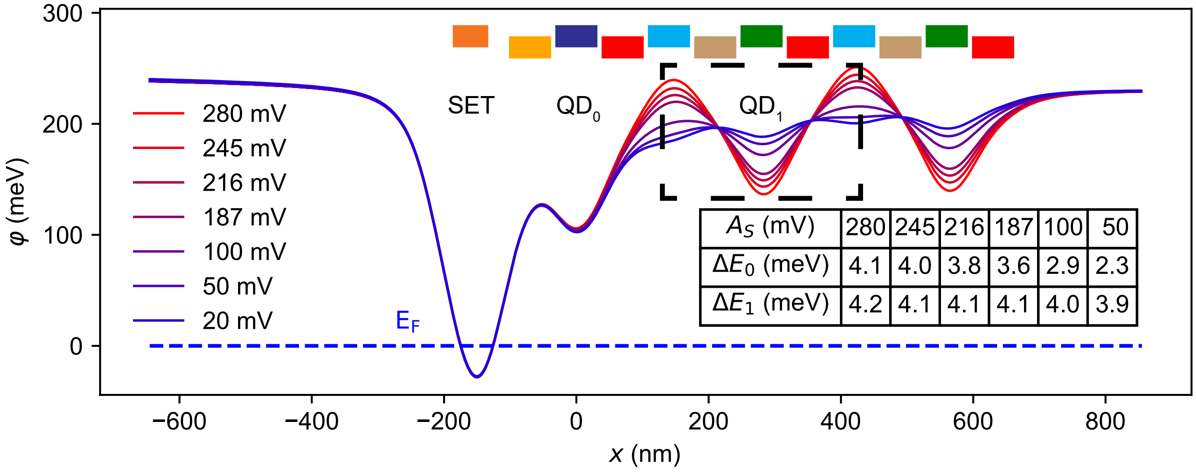

A_S (mV)	280	245	216	187	100	50
ΔE_0 (meV)	4.1	4.0	3.8	3.6	2.9	2.3
ΔE_1 (meV)	4.2	4.1	4.1	4.1	4.0	3.9

Supplementary Figure 4. **Simulated electrostatic potential of 1DEC.** Line-cut of the finite-element numerically simulated electrostatic energy $\varphi(x)$ along the center of QuBus 1DEC for various A_S in the area of the left SET and $QD_{0...2}$. The finite-element model includes the realistic gate-electrode pattern and material layer stack, but no charge defects. Thomas-Fermi approximation is used to simulate the SET in order to take screening of electron reservoirs into account. The Schrödinger-Poisson equation is solved for QD_1 in the region marked by the black dashed rectangle. Three lowest orbitals s, p_x and p_y are used to calculate the lowest orbital splittings ΔE_0 and ΔE_1. The dashed horizontal line represents the Fermi energy E_F for electrons confined by the Si/SiGe heterostructure at operating temperature. Cross sections of SET top-gate and clavier gates are shown on top of the simulated potential using the same color scheme as in Fig. 1a in the main text.
