# Supplementary material for: Si/SiGe QuBus for single electron information-processing devices with memory and micron-scale connectivity function
Source: Nat Commun. 2024 Mar 14;15:2296. doi: 10.1038/s41467-024-46519-x (PMC10940717; doi:10.1038/s41467-024-46519-x)
Supplement: Supplementary file 3 — Description of Additional Supplementary Files [file 41467_2024_46519_MOESM3_ESM.pdf]

File name: Supplementary Video 1

Description: Animated simulation of the conveyor-mode shuttling process. The relative change of the electrostatic potential during a $(\lambda_{+1})^6$ pulse with A_S=280 mV within the quantum well as a function of the x- and y- direction (y=250 nm is center of the shuttle lane) is plotted and animated linear in time. Note that the movie is played in slow-motion and that the $(\lambda_{+1})^6$ pulse takes 120 ms in the experiment. The electrostatic potential is solved for a finite-element model of the device including all electrostatic gates, but excluding any charged defects. The left outermost QD is part of the SET and the barrier to the shuttle lane is open within the simulation. In the experiment it is mainly closed if no electron should be loaded. The black rectangle indicates the boundary within which the time-independent 2D Schrödinger equation is solved for each frame of the animation. The probability distribution of the resulted ground state of the 1-electron wavefunction is plotted within the box without relation to the color bar used for the electrostatic potential.
