# Supplementary figures and images for: Si/SiGe QuBus for single electron information-processing devices with memory and micron-scale connectivity function
Source: Nat Commun. 2024 Mar 14;15:2296. doi: 10.1038/s41467-024-46519-x (PMC10940717; doi:10.1038/s41467-024-46519-x)

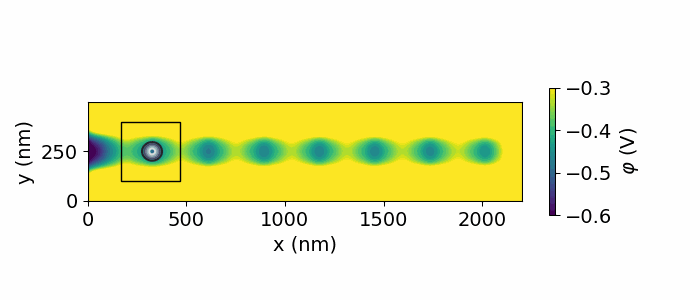

Supplement: Supplementary file 4 — Supplementary Video 1 [file 41467_2024_46519_MOESM4_ESM.gif]
